# Notes upon Diphtheria

**Published:** 1863-02

**Authors:** W. F. Wade

**Affiliations:** London; Senior Physician to the Queen’s Hospital, Birmingham, England


### NOTES UPON DIPHTHERIA.

BY W. F. WADE, M. B., M. R. C. P., LONDON.

Senior Physician to the Queen's Hospital, Birmingham, England.

Four years ago I published a fragmentary memoir upon diphtheria, intending to finish it at an early date. But much remains yet to be done before a complete account of this disease shall be possible. The fact that a great majority of cases occur in private practice, where facilities for minute observation during life are scanty, and post-mortem examinations are constantly refused, is one principal cause of our deficient knowledge. Another is, that public attention has not yet been sufficiently attracted to certain points, the determination of which is essential to any satisfactory history of the disease. In the hope of procuring for these points that investigation which is due to them, and which most assuredly they will eventually obtain, I venture to submit the following propositions to the profession. The style adopted is certainly open to the imputation of curtness; but it seems to me that by divesting the subject as far as possible of extraneous matter and verbiage, those who desire to do so will the more readily arrive at my meaning. I have abstained from particularizing the data on which these conclusions are based. Some of them are received medical dogmas. With regard to the others, the continued prevalence and fatality of diphtheria will enable every one to judge for himself whether or no it presents the features and phenomena here indicated, and whether the practical conclusions here drawn are wholly, partially, or not at all justifiable. I have only to add that, in the hope of concentrating attention upon certain points in the natural history of the disorder, many others of great interest have been entirely omitted.

1.—At the commencement of the present epidemic, being dissatisfied with previous post-mortem examinations, which had been limited to an investigation of those parts whose tissues are continuous with those of the throat, and having noted phenomena which were not thereby explained, I determined, when opportunity should offer, to examine the state of other organs whose tissues were not so continuous.

2—The first post-mortem furnished me with kidneys (of which I retain a drawing) as much altered in appearance as any that we find after death from scarlatinal dropsy.

3.—Obvious pathological analogies led me then to suspect that such a condition would be attended with albuminuria during life. The examination, next day, of the urine of a patient under the care of Mr. Robins, showed that it contained albumen. The frequent occurrence of albuminuria in diphtheria has since been universally recognized.

4.—Curiously enough, subsequent dissections have but rarely furnished me with kidneys so conspicuously altered as these first ones. The changes are more commonly microscopical; consisting of crowding and opacity of the epithelium, which is most readily detached and rapidly disintegrates.

5.—Casts of various kinds are to be found in some specimens of the albuminous urine of diphtheria.

6.—This albuminuria and these anatomical alterations of the kidney are important as showing—

(a) That the disease does not spread solely by continuity of tissue, as had been previously believed;

(*b*) That in some cases the disorder has a tendency to migrate; and in such there is more reason to apprehend croup and other complications than in cases where this migratory tendency is not apparent.

7.—Albuminaria as a symptom of disease is important from the fact of its being frequently, though not necessarily, connected with and dependent upon conditions which impair the excretory action of the kidneys.

8.—In many cases there are indications of diphtherical albuminuria being so associated.

9.—These indications are: diminution of the urine in quantity; suppression of the lithates; nervous symptoms—as indifference to surrounding objects, somnolence, coma—coincidently with the commencement of the albuminuria, and not referable to any other known cause but the kidney complication.

10.—The commencement of the albuminuria may be attended by an increase of the pyrexia, unexplained by any increase of the local disorder or other efficient cause.

11.—These symptoms are relieved by increased urinary exertion.

12.—Albuminuria is not necessarily attended by any obvious symptoms of an unfavorable character.

13.—An imperfect elimination of urinary elements may be unattended by albuminuria. In one case, and sudden diminution of the urinary secretion without albuminuria was attended by swelling and pain of the carpal joints (rheumatic?) The symptoms described in No. 9 are developed coincidently with this imperfect elimination.

14.—I have not observed the early presence of albumen in the urine which, from the concurrent testimony of trustworthy observers, no doubt frequently occurs. Two explanations of this fact offer themselves. In this first place, most of my cases have been seen in consultation, which is demanded in the majority of cases only when fatal symptoms have already supervened. Secondly, my treatment has long been directed to the prevention of kidney complication.

15.—Apart from its early occurrence, there seems to be a special tendency to albuminuria about the seventh or eighth day, at which time the disorder has a natural tendency to terminate. Under such circumstances it is to be looked upon as a critical phenomenon. It may occur at any period.

16.—Kidney affections commonly precedes other complications, such as croup and purpura.

17.—More exact observations upon the amount of urinary excreta before, during, and after intercurrent albuminuria are much wanted. Also in cases where albuminuria does not occur.

18.—If there be retention of urinary elements in the system, it is probable that it tends to induce other complications. (See Dr. Parkes' Lectures upon Pyrexia.)

19.—I have found specimens (of which I retain drawings) of anatomical alterations of the spleen, which has in some instances been found solidified, and of a pinkish-buff color.

20.—The microscope showed that in such spleens there was an unorganized, hyaline, semi-solid material filling the interspaces of the trabeculæ.

21.—I have also found alterations of the spleen, such as Dr. Habershon has described as occurring in cases of purpura.

22.—In no case has manifested alteration of the spleen been found after death where purpura had not been observed during life.

23.—Some cases of purpura have been seen in which I could not undertake to say that the spleen was abnormal.

24.—There is no constant proportion between the severity of the purpuric symptoms and the amount of splenic change.

25.—The vast majority of fatal cases have presented croupy symptoms in the last stage, but many would probably have been fatal without the croup.

26.—In no case that I have dissected was the laryngeal exudation continuous with the faucial.

27.—In no case of croup that I have dissected has the exudation failed to extend beyond the bifurcation of the trachea. In most instances it has extended into the minute ramifications of the bronchi.

28.—The tracheal and bronchial exudation has varied in consistency from a very firm membrane to a pasty granular layer.

29.—In two cases, besides (other?) purpuric symptoms, I found after death nodules of pulmonary apoplexy.

30.—In one case I thought that there was some hyaline exudation in the supra-renel capsules. In that case, and in another, these organs were intensely vascular.

31.—We are justified by the preceding observations,as well as by other well-known symptoms of the disease, in looking upon diphtheria as a zymotic disease; not as Bretonneau conceived it to be, a local disease, spreading by continuity of tissue, and only affecting the system in a secondary manner.

32.—I have never stated, and I am not prepared to state, my opinion upon the relation, if any, between diphtheria and scarlatina.

33.—To those who find less difficulty in coming to a positive conclusion upon the point, I beg to recommend the following considerations:

(*a*) Scarlatina and diphtheria may be associated.

(*b*) Scarlatina is not necessarily accompanied by efflorescence, or by noticeable fever.

(*c*) Diphtheria may probably affect the system without producing any throat exudation.

(*d*) Scarlatina may recur.

(*e*) Certain forms of scarlatina may be *accompanied* by albuminuria.

(*f*) Scarlatinal albuminuria does not necessarily produce dropsy; dropsy, in fact, is the exception in albuminuria *accompanying* scarlatina.

(*g*) Any occasional form of a specific fever may become the type of an epidemic.

(*h*) Granting that scarlatina and diphtheria are both zymotic disorders, we do not know what is the nature of their respective poisons.

34.—Local treatment exerts no known influence upon the general course of specific fevers.

35.—The true rule of practice in such diseases is to obviate the tendency to death.

36.—The tendency to death in diphtheria is sometimes by asthenia, directly induced by the blood-poison; sometimes by complications, of which the earliest is generally a kidney affection, interfering with urinary elimination. We must therefore eliminate the poison, and if possible *prevent* the complications.

37.—In pyrexial disorders, one of the most constant and mysterious phenomena is the quantity of water disposed of by the system. (See Parkes on Pyrexia.)

38.—In diphtheria the quantity of ingesta will be commonly small if the patient be allowed to consult his own convenience.

39.—Water is essentially necessary to the performance of the urinary functions.

40.—Concentration of the urine is equivalent to kidney irritation.

41.—Diphtheritic albuminuria is often preceded by urine of high specific gravity. The supervention of albuminuria may fail to produce this.

42.—It is often preceded by deposit of lithates, showing a comparative paucity of the urinary water.

43.—All plans of treatment which have been adopted on the large scale for the treatment of diphtheria have embraced the ingestion, in large quantities, of fluid nutriment as an important if not essential element.

44.—By the copious administration of pure water or diluents in diphtheria, the urine may be enormously increased in quantity, often without corresponding diminution of its specific gravity.

45.—This seems to indicate that the detritus of interstitial metamorphosis had been previously insufficiently eliminated.

46.—I recommend the ingestion of bland fluids in as great quantity as the patient will take; half a pint every hour or two, if possible, in the case of adults.

47.—To avoid chills, I recommend that in all cases the patients should be clothed from head to foot in a flannel gown, and kept in bed. I believe that the adoption of this plan would have saved almost innumerable lives.

48.—Assuming the presence of a substantive poison in the system we know no drug which will act as a direct eliminant but iodide of potassium. It positively eliminates lead, and we may presume that it positively eliminates syphilis.

49.—I employ iodide of potassium in two, three, or four grain doses every two or three hours. I have been in the habit of conjoining with it chlorate of potass in five to ten grain doses.

50.—I have known no instance of a fatal termination where this plan of treatment had been carried out. I have known no instance of serious symptoms or of secondary paralysis supervening where this plan had been rigorously carried out. The difficulty, especially with children, is in ensuring a copious supply of fluid.

51.—This plan exercises a speedy and salutary influence upon the general symptoms of the disease. The exudation often diminishes with extraordinary rapidity. Essential fevers run a definite course, and can be rarely if ever cut short. Till the disease has gone we cannot be free from the danger of complication. Hence the immense importance of continuing the treatment after immediate relief has been obtained.

52.—Aqueous injections may be employed to supplementalize ingestion by the mouth; but this is a plan of very inferior efficacy. If deficiency of urine be present, bitartrate of potash, sinapisms to the loins, warm bath, and solution of acetate of ammonia help to restore it.

53.—This general plan of treatment does not preclude other remedies in special cases, or to meet special indications.

54.—Where it has been carried out I have not found a necessity for stimulants, nor have I found that these, when administered, have produced that immediate and sensible (even if incomplete) amelioration that we expect to see in cases where they have a beneficial influence.

55.—The same may be said of tonics and iron. I have never met with such marked anatomical alterations as in cases which had been freely treated with a mixture containing muriated tincture of iron and hydrochloric acid. It does not necessarily follow from this that such remedies may never be required; but they should not be used indiscriminately and recklessly.

56.—It is contrary to the ordinary rules of our art to interfere with the local development of blood-poisons, except for special reasons.

57.—The faucial exudation of diphtheria is to be considered as the local manifestation of a general disease.

58.—Interference with it will not prevent its reproduction, nor will it prevent laryngeal complication, nor will it prevent the supervention of grave constitutional disorder. It is exceedingly irksome to young patients.

59.—We are justified in interfering with the throat exudation when there is excessive fetor, or when it is so copious as to interfere with respiration or deglutition—not otherwise.

60.—If the croup always extend below the bifurcation of the trachea, tracheotomy is but a forlorn hope; as such it may be right to resort to it in some cases.*

61.—I am not satisfied with that explanation of the secondary paralytic affections which attributes them to reflex irritation. Possibly minute dissection might discover some organic change in (*a*) the nervous centres, (*b*) the nervous periphery, or (*c*) the muscular tissue.

62.—Albuminuria may or may not be present in cases of diphtheritical paralysis.

63.—Cases of paralysis progress so slowly when treated simply by quinine and other tonics as to lead to the supposition that these drugs exert

* According to M. Roger, twenty per cent. of the children operated upon at the Hospital des Enfans Malades in Paris recover.—*Archives Generales de Medicine, April*, 1862.

no direct influence upon this sequela, which probably in such cases wears itself out.

64.—I believe that I have obtained more speedy results with eliminants—as iodide of potassium, iodide of iron, and bichloride of mercury with bark.

65.—Blisters to the top of the sternum, if applied early, seem to exercise a most beneficial influence upon the paralysis of the palate.

66.—Paralysis may follow, as kidney complication may attend, slight as well as severe cases of diphtheria. In one case I have heard that the paralysis has lasted two years, and may be considered permanent.—*London Lancet.*

